# How Two-Child Policy Affects China's Energy Consumption: The Mediating Role of Lifestyle

**DOI:** 10.3389/fpubh.2022.866324

**Published:** 2022-04-06

**Authors:** Fengzhang Chen, Wei Wang, Yanfei Wang, Yongqiu Wu

**Affiliations:** ^1^College of Computer Science, Chongqing University, Chongqing, China; ^2^School of Economics and Business Administration, Chongqing University, Chongqing, China; ^3^Party School of CPC Chongqing Municipal Committee, Chongqing, China

**Keywords:** two-child policy, lifestyle, energy consumption, fertility rate, scenario analysis

## Abstract

**Background:**

Existing literature believed that the birth control policy affects energy consumption through the change in population size, but ignored the changes in people's lifestyle. This may mislead the government's policy-making about population and energy consumption.

**Method:**

This article proposed a Population-Lifestyle-Energy (PLE) model to provide new insights into how birth control policy affects energy consumption if the changes in people's lifestyle are considered. The ProFamy software is used to forecast the changes in demographic characteristics. The methods of regression analysis and Input-Output Analysis are used to predict the impacts of lifestyle changes on energy consumption.

**Results:**

We find that China's two-child policy will result in the total energy consumption increase by 16.2% in 2050, far outpacing the population increase of 9.3% when considering the indirect effect of lifestyle changes. This is significantly different from the optimistic wisdom in the existing literature. We also find the non-linear relationships between fertility rate and energy consumption.

**Conclusion:**

Ignoring lifestyle changes will lead to an underestimation of energy consumption. Contrary to conventional optimistic wisdom, we believe that the two-child policy will make it difficult for China to meet promised energy conservation goals.

## Introduction

The mounting need for energy use portends hazardous consequences on human health (1). The population is one of the most important factors that determine energy consumption (2, 3). China is the most populous and the most energy-consuming country in the world (4). After the implementation of the one-child policy in the 1970's, the birth rate continued to fall, far below the world average. Therefore, many studies believed that the declining fertility rate would lead to a decrease in energy consumption in China (5, 6). However, the Chinese new birth control policy is changing the trend of population growth. In 2010 (the last year of the one-child family policy), the birth rate was only 11.9 per thousand, and the second child and above accounted for 44.1% of newborns. In November 2011, China implemented a two-child fertility policy for couples where both the husband and the wife are from a single-child family. In January 2016, China replaced the one-child policy with the universal two-child policy. In 2020, there were 12.02 million newborns in China, of which the second- or multi-child accounted for 57.6%. Although the two-child policy has made a significant increase in the fertility rate and population size, few works of literature have concerned the potential challenges posed by the two-child policy to China's energy consumption (7).

Many works of literature have studied the impact of population size decided by the fertility rate on energy consumption (8–13). However, these studies had an obvious defect, as they were based on the assumption of “representative households/individuals,” i.e., all individuals and households have the same lifestyle and consumption pattern (14–16). In other words, the traditional assumption focused on the change in population size but unfortunately ignored the changes in people's lifestyle (17, 18). Actually, the fertility rate not only changes population size but also changes the people's lifestyle (e.g., household size, consumption pattern, and travel style) (19–21). The changes in lifestyle will have an important impact on energy consumption (16, 22). For example, residential energy consumption (REC) is decided by both the household number and household type^1^ (12, 21, 23). Because the fertility rate will not change the number of households in the short run, only the household type (21), the REC will be mis-estimated if the changes of household type are ignored.

The existing literature has focused on the effect of population size on energy consumption and ignored the mediating effect of lifestyle changes (8, 9, 12, 24, 25). This article tried to comprehensively analyze the impact of birth control policy on energy consumption by considering the changes in the population size and people's lifestyles. We find that lifestyle changes cannot be ignored in energy consumption forecast, and the impact of fertility rate on lifestyle and energy consumption varies across end-users. Firstly, the two-child policy will change the household size in the residential sector. The traditional literature classified household types by the intergenerational structure (3, 16, 26). However, the two-child policy will not change the intergenerational structure in the household in the short term but will increase the household size.^2^ Therefore, the impact of the two-child policy on the REC can only be found through the changes in household number and household size. Secondly, the two-child policy will change the quantity and structure of the demand for goods and services, because of the different lifestyles in the different life cycle stages. Then energy consumption in the industrial and commercial sectors and freight transport sector will change accordingly. Thirdly, the travel style is different for the people in the different life cycle stages. We will study the impact of travel style on energy consumption in the passenger transport sector, which was neglected in traditional works of literature (27, 28). Therefore, the impact of the two-child policy on energy consumption is not equal to the population growth when considering the changes in lifestyle. The findings of this study indicate that the two-child policy will change the trend of China's energy consumption and significantly impact the Chinese government in achieving the promise of energy conservation and emission reduction goals. We believe that the traditional view that the slowing population growth will reduce China's energy consumption in the future is overly optimistic (4, 8, 24). This article is among the first to reveal the transmission mechanism between fertility rate and energy consumption by simultaneously considering the dynamic changes of lifestyle and population size. This study has important implications for energy consumption forecasting and formulating the energy conservation policies of the governments and international organizations.

## Objectives and Scenarios

**Figure 1:**
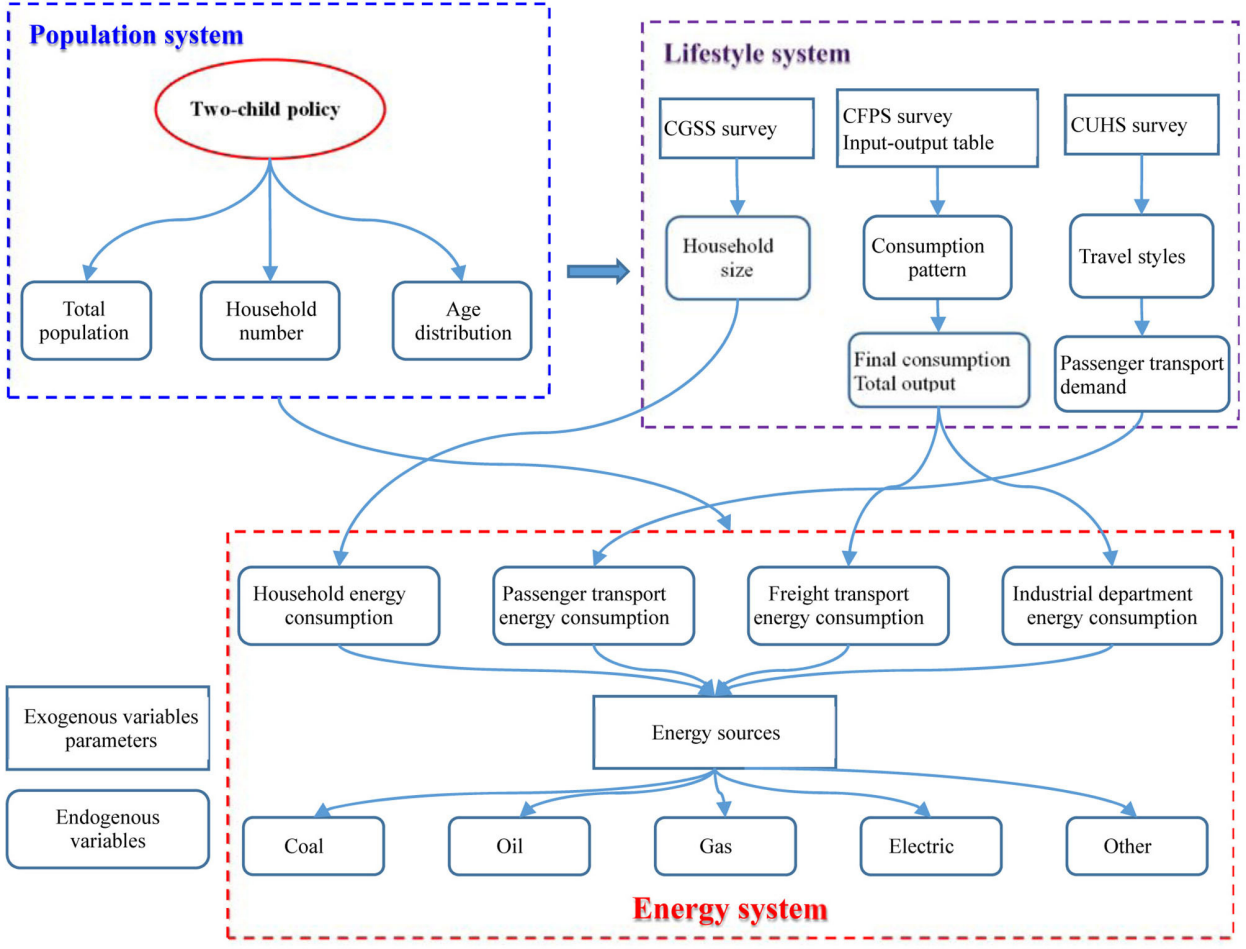
Population-Lifestyle-Energy (PLE) model: the impact of the two-child policy on energy consumption.

The target of this study was to quantify the impact of birth control policy on energy consumption in China. To address the aforementioned shortcomings of traditional research, this article presents a Population-Lifestyle-Energy (PLE) model to evaluate the impact of the fertility rate on energy consumption (Figure 1). Many works of literature have concerned the impact of the changes in consumer lifestyle on energy consumption (16, 29); etc.]. These studies mainly focused on the individual energy consumption (30), but ignored the effects on the enterprise sector (31). This study analyzes the mediating effects of lifestyle changes on energy consumption from three perspectives: household size, consumption structure, and travel mode. Compared with the traditional models (11), the PLE model will provide new insights into how fertility rate affects energy consumption by combining the micro survey data with the macro statistical data and reveals quantitative relationships among fertility rate, the changes of lifestyle, and energy consumption for different sectors.

In the existing models, there are different classifications for the end-user of energy. The most popular classification includes four sectors, i.e., residential, commercial, transportation, and industrial sectors [9; 10; etc.]. Since the industrial and commercial sectors used similar data and methods to predict the energy consumption in the PLE model, they were combined into one sector. Because the energy consumption in passenger transport and freight transport is predicted using different methods, the transport sector is divided into two sectors. Therefore, the end-user of energy is decomposed into four sectors, i.e., residential, industrial and commercial sector, passenger transport, and freight transport sector. This classification is consistent with the Structure of Extended Snapshot Tool (ExSS) (17).

The birth control policy produces a “direct effect” on the residential and passenger transport sectors. In the residential sector, the PLE model abandons the assumption of standardized household and simultaneously considers the changes of the household number and household size (21, 26). In the passenger transport sector, the two-child policy will affect the passenger transport demand and its structure due to the differences in travel mode among different life cycle stages (28).

The birth control policy produces an “indirect effect” on the industrial and commercial sectors and freight transport sector. In the industrial and commercial sectors, the two-child policy will change the consumption pattern, thus affecting the demand for goods and services. Then the energy consumption can be estimated by the Input-Output Table Analysis (19, 20, 22). Energy consumption in the freight transport sector is decided by the goods and service demand of the industrial and commercial sectors (32) and is also estimated by the Input-Output Table Analysis.

In the PLE model, the first step of the energy demand forecast is to predict the demographic characteristics. China has implemented the universal two-child policy since 2016. The new birth control policy would lead to a sharp increase in the number of two-child births (7). To assess the impact of the new policy on energy consumption, we simulate two different variations that assume different fertility policies.

### Scenario I

The old birth control policy (one-child policy). Each couple can have only one child. According to the *Chinese Population and Family Planning Law*, each illegal extra child would be fined 3–10 times of his/her annual income. In 2010, the total fertility rate in the rural areas was 1.80, with the fertility rates of one-child, two-child, three-child, and more being 0.943, 0.675, and 0.164, respectively; the total fertility rate in the urban areas was 1.22, with the fertility rates of one-child, two-child, three-child, and more being 0.854, 0.320, and 0.046, respectively. In Scenario I, we assume that the total fertility rate and the distribution in urban and rural areas remain constant.

### Scenario II

The new birth control policy (two-child policy). Under the new birth control policy, every couple can have two children. Due to the concentrated outbreak of the accumulated fertility desire, the two-child fertility rate was very high in the early stage of the implementation of the policy. In 2019, the number of two-child births exceeds the number of one-child births. Therefore, population projections based on fertility rates in recent years are unreliable. Referring to the survey results of fertility preference (33), we make the following assumption in Scenario II: (1) the two-child policy will not affect the first child fertility rate; (2) the total fertility rate in the rural areas is 2.140, with the fertility rates of one-child, two-child, three-child, and more being 0.943, 0.963, and 0.234, respectively; (3) the total fertility rate in the urban areas is 1.808, with the fertility rates of one-child, two-child, three-child, and more being 0.854, 0.834, and 0.120, respectively.

The following discussion concentrates on the impact of the two-child policy on population characteristics and energy consumption, drawing comparisons with the scenario of the one-child policy remaining in place. Since the two-child households are formed from the one-child households, the new birth control policy will not change the intergenerational structure of households in the short term, but only change the household size. The two-child policy will only lead to an increase in the household number when the new births grow up and leave the parental home. In the scenario prediction, we take 2016 as the benchmark year, when China began to implement the universal two-child policy. Being the final year of our analysis 2050, people born after 2016 will come of age. Although we have not analyzed the trend across the life cycle, this study can provide effective evaluation for the influence of the two-child policy and the knowledge to guide policy-making.

The ProFamy software is used to predict the changes in demographic characteristics during 2016–2050 (34)^3^. The original data and model parameters are derived from the sixth census and the annual sampling survey data,^4^ including probabilities of surviving, marital status transitions, age-specific fertility frequencies, life expectancies at birth, mean ages at birth, mean ages of children leaving the parental home, proportions of elderly living with children, and sex ratio at birth. The data of future urbanization rates are collected from the Human Development Report 2019 (35).

## Methodology

The energy consumption of four sectors, namely, the residential sector, industrial and commercial sectors, passenger transport sector, and freight transport sector, are predicted using different methods and data.

### Model of the Residential Sector

Referring to the existing references (36), the number of the standardized households for different energy types is calculated by the following equation:


(1) $$\begin{array}{r} THI_{et,reg}=\overset{7}{\underset{ht=1}{\sum }}\left(HN_{ht,reg}\times HI_{et,reg,ht}\right), \end{array}$$

Where,

*et* is energy type including coal, oil, natural gas, electricity, and other energy sources.

*reg* is regions (including urban and rural). There are different energy consumption patterns between urban and rural residents in China (16).

*ht* is household type divided into 7 groups according to the persons per household: 1–6 persons and more than 7 persons.

*HN* is the number of household, which is predicted by the ProFamy software (34). The parameter assumption has been introduced in Section Objectives and scenarios.

*HI* is the energy consumption index for different household types. The data are calculated using the Chinese General Social Survey (CGSS) database (Supplementary Table 1)^5^. The one-person household is assigned as the standard household (*HI* = *100*). The household energy consumption does not increase in proportion to the persons per household. The larger the household size, the lesser the energy consumption per person. Other types of households can be converted to the standard households according to the *HI*.

*THI* is the total number of standardized households, which is classified by the different energy types.

The calculation formula of total energy consumption in the residential sector is


(2) $$\begin{array}{r} REC_{et}=\underset{reg}{\sum }\left(THI_{et,reg}\times AHI_{et,reg}\right), \end{array}$$

Where,

*AHI*_*et, reg*_ is the average energy consumption for a standardized household. The *AHI* is predicted by Logistic Model on the basis of historical data except for the coal consumption in the rural regions (Supplementary Figure 1). Due to the accelerated construction of the natural gas pipelines, the use of natural gas will significantly increase in the future (37). Due to the popularity of household electrical appliances, the electricity consumption will also significantly increase (38). Because of the stricter environmental policy and wide use of home appliances (4), the consumption of coal in the future Chinese families will continue to decline. Therefore, the *AHI* of coal in the rural region is predicted by the Linear-Quadratic Model.

*REC*_*et*_ is the residential energy consumption for different energy types. It is the sum of the rural and urban regions.

### Model of Industrial and Commercial Sectors

Firstly, resident's demand for goods and services is calculated according to the population size and per capita consumption. Considering the changes of population size and structure, the growths of consumption expenditure in different industrial and commercial sectors are


(3) $$\begin{array}{r} GDC_{reg,id,t}=\frac{\sum_{life}POP_{life,reg,t}\times CON_{life,reg,id}}{\sum_{life}POP_{life,reg,2017}\times CON_{life,reg,id}}, \end{array}$$

Where,

*id* denotes the goods and service categories, which are divided into eight aspects (16): (1) food, tobacco, and alcohol, (2) clothing, (3) housing, (4) facility and services, (5) communication and transportation, (6) education and entertainment and cultural activities, (7) healthcare, and (8) miscellaneous goods and services.

*t* is the year.

*life* is life cycle stages, including minors, adults, and elders.^6^ People's lifestyles are different in different life cycle stages.

*POP* is the population size, which is predicted by the ProFamy software (34).

**Figure 2:**
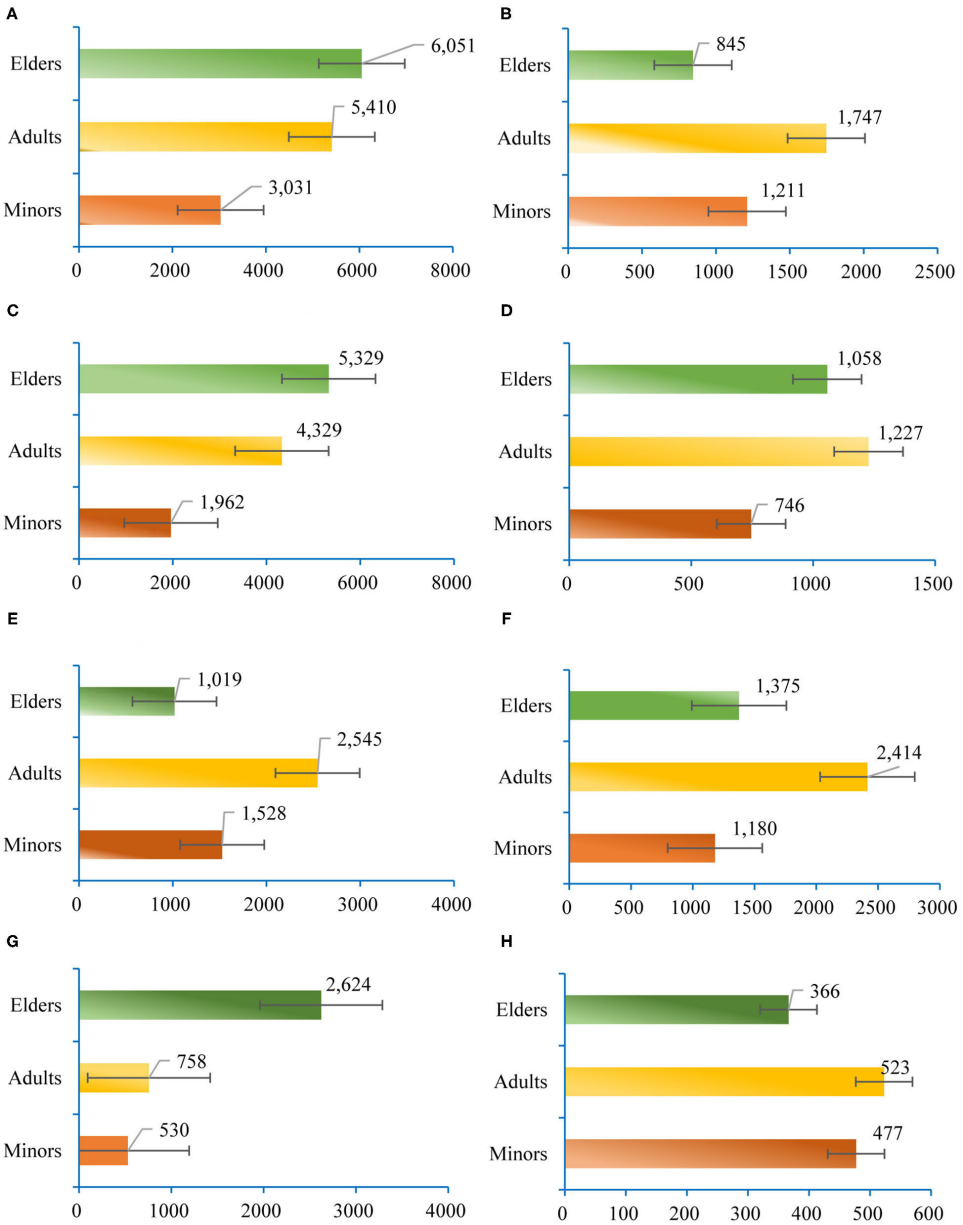
Demand for goods and services in different life cycle stages (Unit: CNY). **(A)** Expenditure on food, tobacco, and alcohol. **(B)** Clothing expenditure. **(C)** Housing expenditure. **(D)** Expenditure on facility and services. **(E)** Expenditure on communication and transportation. **(F)** Expenditure on education and entertainment, and cultural activities. **(G)** Healthcare expenditure. **(H)** Expenditure on miscellaneous goods and services.

*CON* is the index of consumption expenditure per capita for different life cycle stages. The data are derived from the China Family Panel Studies (CFPS) database^7^. Minors have less consumption expenditure than other groups for the most goods and services. The elders have the fewest expenditure on clothing, communication, and transportation (Figure 2).

*GDC* represents the growth of consumption expenditure caused by the demographic changes (Supplementary Figure 2). Affected by urbanization (11), the consumption expenditure in the rural regions will decrease, while the urban regions will increase. Due to the changes in demographic characteristics, residential consumption expenditure on communications, transportation, and clothing grew slowly, but healthcare expenditure is the fastest growing.

Then, the consumption expenditure of urban and rural regions is calculated by,


(4) $$\begin{array}{r} GCI_{reg,id,t}=\frac{WP_{reg,t}\times GPW_{reg,t}\times CP_{reg,id,t}}{WP_{reg,2017}\times GPW_{reg,2017}\times CP_{reg,id,2017}}, \end{array}$$

(5) $$\begin{array}{r} GHC_{reg,id,t}=GDC_{reg,id,t}\times GCI_{reg,id,t}, \end{array}$$

Where,

*WP* is the working-age population, which is predicted by the ProFamy software.

*GPW* is GDP per-worker, which is estimated according to the EIA prediction (10) (Supplementary Table 2).

*CP* is the propensity to consume, which is estimated according to the experience of European Union Nations (39) (Supplementary Table 2).

*GCI* is the growth of consumption expenditure caused by the income increase. It represents the influence of exogenous economic growth.

*GHC* is the growth of household consumption expenditure (HCE), including rural regions and urban regions. It represents the total growth of consumption expenditure caused by demographic changes and economic development.

Secondly, the method of Input-Output Analysis is used to estimate the energy demand in the industrial and commercial sectors (38). China's Input-Output Table subdivided goods and services into 149 sectors. The most recent table in 2017 is used to calculate the total output and intermediate input of each sector. Government consumption expenditure (*GCE*) and total capital formation (*TCF*) are assumed to increase year-on-year with the residents' final use, regardless of the changes in imports and exports. The *HCE* is calculated as


(6) $$\begin{array}{r} HCE_{reg,se,t}=HCE_{reg,se,2017}\times GHC_{reg,se,t}, \end{array}$$

Where,

*reg* represents the rural or urban region.

*se* represents 149 sectors in the Input-Output Table. Because *GHC* is classified into 8 categories, the value of *GHC*_*reg, se, t*_ is assigned according to the categories (*id*) to which each sector (*se*) belongs.

According to the final use matrix and the consumption coefficient table of input product, the final total expenditure is calculated by


(7) $$\begin{array}{r} TI_{t}=\left(HCE_{t}+GCE_{t}+TCF_{t}\right){\times \left(I-CCM\right)}^{-1}, \end{array}$$

Where,

*HCE* is the sum of household consumption expenditure in urban and rural regions.

In Equation (7), *HCE, GCE*, and *TCF* are all in vector forms of 149 sectors.

*I* is 149^*^149 unit matrix.

*CCM* is a consumption coefficient matrix and is assumed to remain constant.

*TI*_*t*_ is the total input vector for 149 sectors in the future (Supplementary Figure 3).

Finally, the consumption of various energy types is estimated. Because the Input-Output Table is the monetary measurement, the energy consumption in physical measurement can be derived from:


(8) $$\begin{array}{r} ECI_{et,t}=\frac{TI_{et,t}}{TI_{et,2017}}{\times ECI}_{et,2017}, \end{array}$$

Where,

*TI*_*et, t*_ is the total input for five types of energy including coal, oil, natural gas, electricity, and other energy sources. The data of *TI*_*et, t*_ are extracted from *TI*_*t*_ according to the corresponding sectors.

*ECI*_*et, t*_ is energy consumption for energy type *et*.

### Model of Passenger Transport Sector

First, the impact of population size and lifestyle changes on the passenger transport demand is estimated by:


(9) $$\begin{array}{r} PDC_{vt,reg,t}=\frac{\sum_{life}POP_{life,reg,t}\times TEI_{life,reg,vt}}{\sum_{life}POP_{life,reg,2017}\times TEI_{life,reg,vt}}, \end{array}$$

Where,

*vt* is vehicle type for passenger transport, such as railway transport, road transport, waterway transport, and air transport.

*TEI* is the travel expense index for various vehicle types. People in different life cycle stages, namely, minors, adults, and elders, have different travel modes. The data are derived from the Chinese Urban Household Survey (CUHS) database.^8^ The adult group is taken as the control group (*TEI* = 100). The travel expense index of the minor group is significantly lower than that of other groups. The elder group is second only to the adult group (Supplementary Figure 4). Since there is no rural household survey database, we assume that the travel expense index of the rural regions is the same as the urban regions.

*PDC* is the growth of passenger transport demand caused by the changes in demographics and lifestyle, and 2017 is the base year.

Second, assumed that the mileage per capital remains constant, the demand of total passenger transport can be calculated by


(10) $$\begin{array}{r} PK_{vt,reg,t}=PDC_{vt,reg,t}\times PK_{vt,reg,2017}, \end{array}$$

Where,

*PK* is the demand of total passenger transport (passenger-kilometer) for different vehicle types and regions.

Finally, according to the changes of passenger transport demand, the passenger transport energy consumption for various vehicle types is calculated as follows:


(11) $$\begin{array}{r} ECV_{vt,et,t}=\underset{reg}{\sum }PK_{vt,reg,t}\times PET\text{\_}P_{vt,et,t}, \end{array}$$

(12) $$\begin{array}{r} ECP_{et,t}=\underset{vt}{\sum }ECV_{vt,et,t}, \end{array}$$

Where,

*ECV*_*vt, et, t*_ is the energy consumption of energy type *et* in different vehicles.

*PET*_*P*_*vt, et, t*_ is the proportion of energy type *et* consumption in the vehicle *vt*. The data of *PET*_*P* are derived from the Input-Output Table.

*ECP*_*et, t*_ is total consumption of energy type *et*.

### Model of Freight Transport Sector

Using the data of the total input vector (*TI*), which is predicted in Equation (7), the freight transport demand for various transport tools can be calculated by


(13) $$\begin{array}{r} FDT_{ftt,t}=\underset{se}{\sum }TI_{se,t}, \end{array}$$

Where,

*ftt* is freight transport tools, including railway freight transport, road freight transport, water cargo transportation, air cargo transport, and other transport tools.

*se* denotes the sectors in *TI* corresponding to the freight transport tools. The railway freight transport matches with the sector of “railway freight transport and transport support activities;” the road freight transport match with the sector of “road freight transport and transport support activities;” the water cargo transportation match with the sector of “water cargo transportation and transportation support activities;” the air cargo transport match with the sector of “air cargo transport and transport support activities;” the other transport tools match with the following sectors: “pipeline transportation,” “multimodal transport and transport agents,” “handling and storage,” and “postal service.”

*FDT* is the freight demand for different transport tools.

Then, the energy demand of each energy type and transport tools is calculated by


(14) $$\begin{array}{r} FDE_{ftt,et,t}=FDT_{ftt,t}\times {FET\text{\_}P}_{ftt,et,t}, \end{array}$$

Where,

*FET*_*P*_*ftt, et, t*_ is the proportion of energy type *et* consumption in the freight transport tool *ftt*. The data are derived from the Input-Output Table.

*FDE* is the energy consumption of the different energy types and freight transport tools.

Finally, the energy consumption of each energy type *et* in the freight transport sector is


(15) $$\begin{array}{r} ECF_{et,t}=\underset{ftt}{\sum }\frac{FED_{ftt,et,t}}{FED_{ftt,et,2017}}{\times ECF}_{ftt,et,2017}. \end{array}$$

## Results

### Impact of the Two-Child Policy on Population and Lifestyle

**Figure 3:**
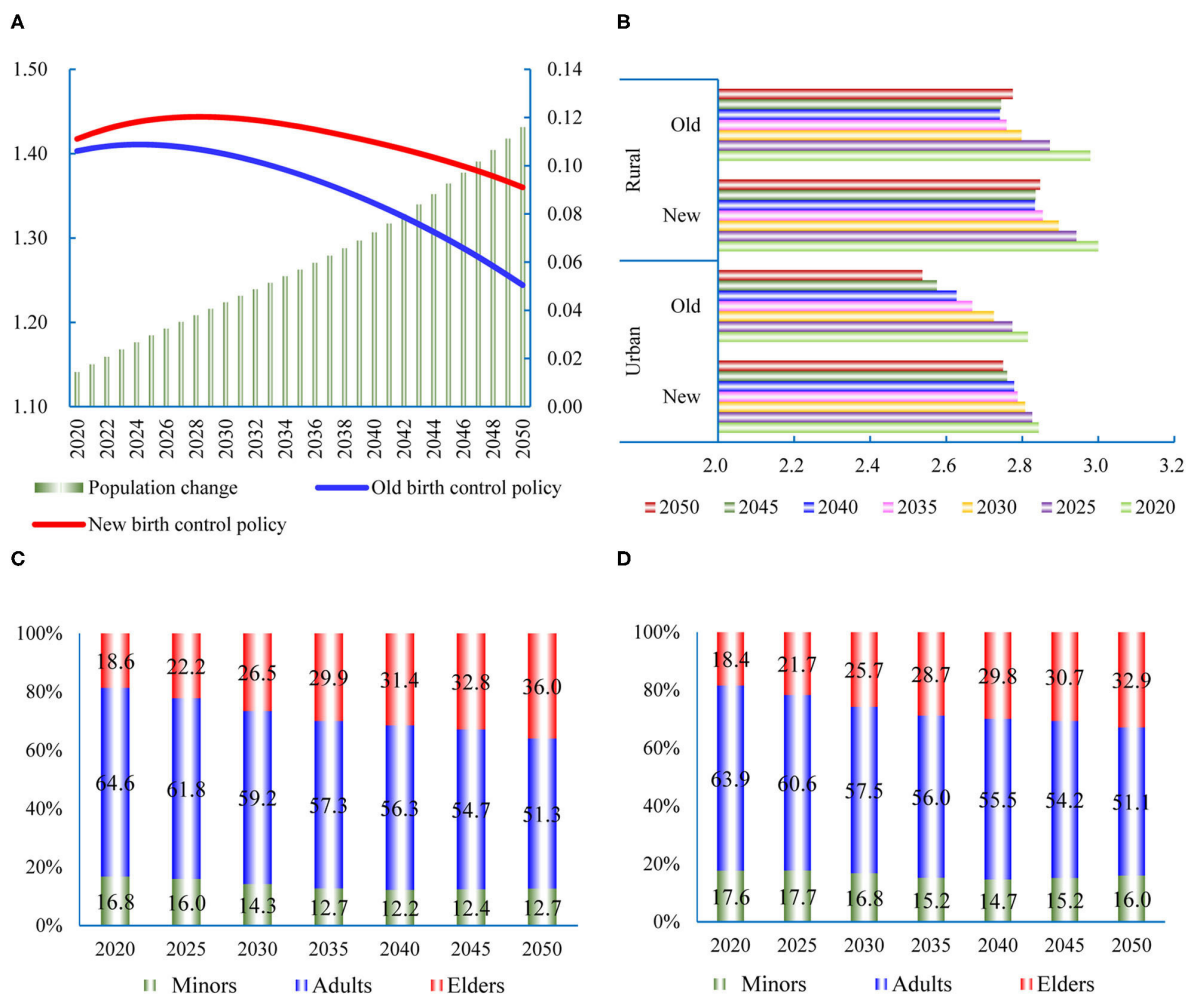
Changes of population characteristics and household size. **(A)** The changes of population size (billion persons). **(B)** Average household size (persons). **(C)** Population distribution for different life cycle stages in old policy. **(D)** Population distribution for different life cycle stages in new policy.

The ProFamy software is used to predict the future population (34). If the one-child policy continues in China, the total population size in 2050 is expected to reach 1.244 billion, of which the urban population will be 0.913 billion, and the urbanization rate will be 73.4% (Figure 3A)^9^. These results are close to the forecast of the *Human Development Report 2019* (35). After implementing the two-child policy, the total population is estimated to reach 1.360 billion in 2050, and the urbanization will stand at 74.2%. Compared with the old birth policy, the new policy is estimated to result in an increase of 14.3 million persons in 2020 and an increase of 116.0 million persons in 2050.

Because of the changes in work style and ideology, the size of China's households will slowly decrease in the future (40, 41). However, the two-child policy will delay this trend (Figure 3B). If the two-child policy is not implemented in the urban region, the average household size will slowly drop to 2.54 in 2050. However, after the implementation of the two-child policy, the household size will remain at the level of 2.75. The household size in the rural regions will increase by 0.07 in 2050, which is smaller than that in the urban regions. The main reason is that the Family Planning Law has not been strictly abided in the rural region. The two-child policy will change the population in different life cycle stages (Figures 3C,D). In 2050, the new policy will reduce the proportion of elders (over 60 years old) by 3.1% and increase the proportion of minors (under 14 years old) by 3.3%. Many studies confirmed that population aging can reduce energy use (42). We can infer that the new birth control policy in China will increase the energy consumption per capita because of the decline of the elderly population proportion. It will increase the challenge in achieving the target of Chinese carbon emission.

### Energy Consumption in the Residential Sector

**Figure 4:**
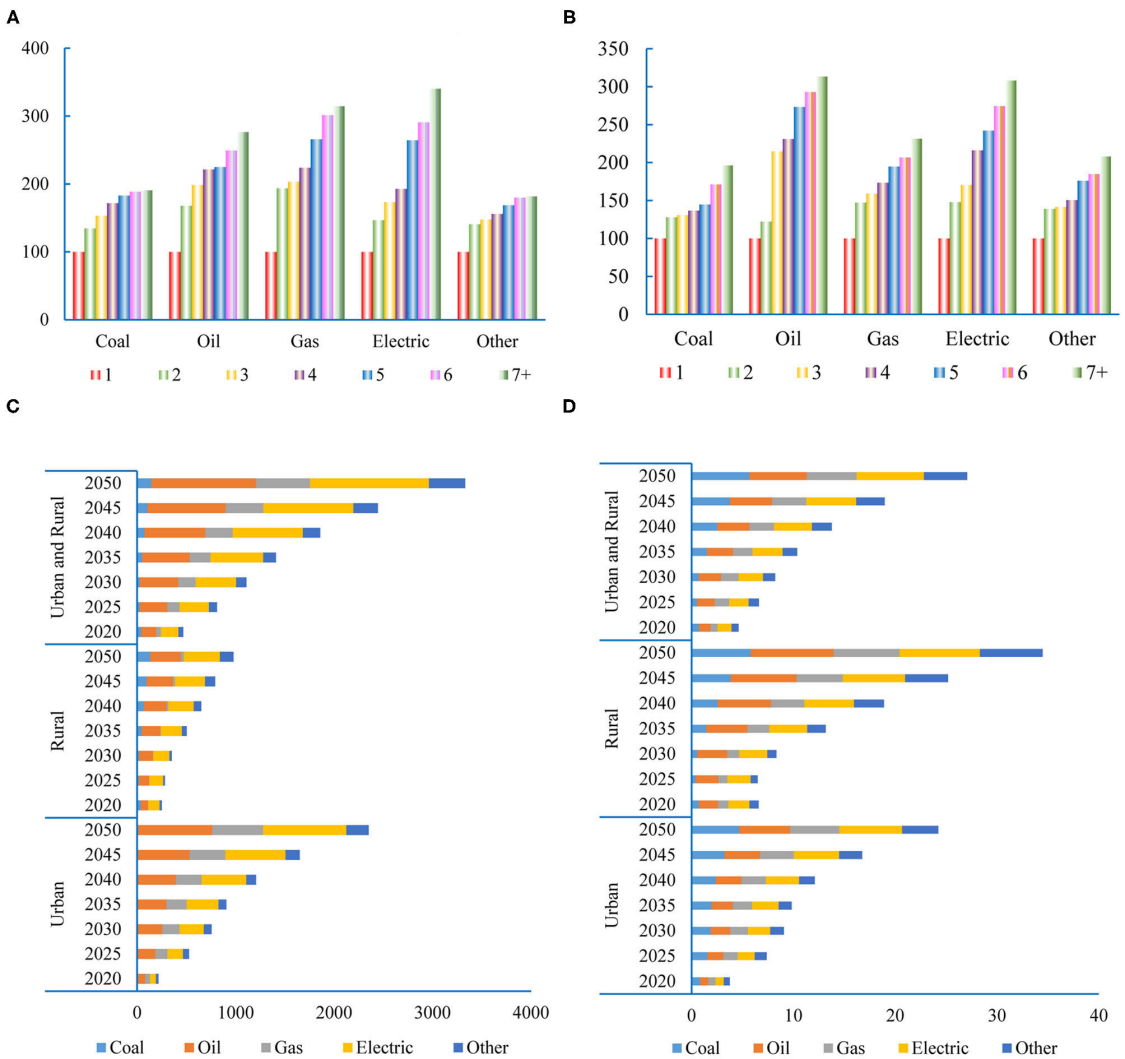
Impact of the two-child policy on residential energy consumption (REC). **(A)** Energy consumption index for different household types (urban,one person household = 100). **(B)** Energy consumption index for different household types(rural, one person household = 100). **(C)** The increase of energy consumption caused by the two-child policy (Mtce). **(D)** The rate of energy consumtipn change caused by the two-child policy (%).

In Figures 4A,B, the average energy consumption for different types of households is shown (one-person household is the standard household). These statistics indicate that energy consumption per household is not proportional to household size. It has a significant scale effect that energy consumption per person is declining with the expansion of household size. Taking the electricity consumption as an example, the two-person household is 148.0% of the standard household; the three-person household is 170.5% of the standard household, and the household with more than seven persons is 307.9% of the standard household.

Using the prediction of the household number and household size, we can estimate the REC in Scenario I and II, respectively. The impact of the new birth control policy on REC is small in the short term and will increase over time (Figure 4C). In 2020, when compared to the one-child policy, the new policy will lead to an increase in REC by 4.69 Mtce (the rate of change is 1.01%)^10^; in which urban region will increase by 2.18 Mtce (the rate of change is 0.75%) and rural region will increase by 2.51 Mtce (the rate of change is 2.25%). By 2050, the impact of the two-child policy changes on the resident's energy consumption will reach 33.31 Mtce, an increase of 5.59%, of which urban region will increase by 23.52 Mtce, accounting for 70.6%; rural region will increase by 9.79 Mtce, accounting for 29.4%. The impacts of the two-child policy on different types of energy consumption are shown in Figure 4D. In 2050, the two-child policy will result in a 9.33% increase in population, but the energy consumption will only increase by 5.59%. The reason is that the expansion of household size leads to the decline of energy consumption per capita.

### Energy Consumption in the Industrial and Commercial Sectors

**Figure 5:**
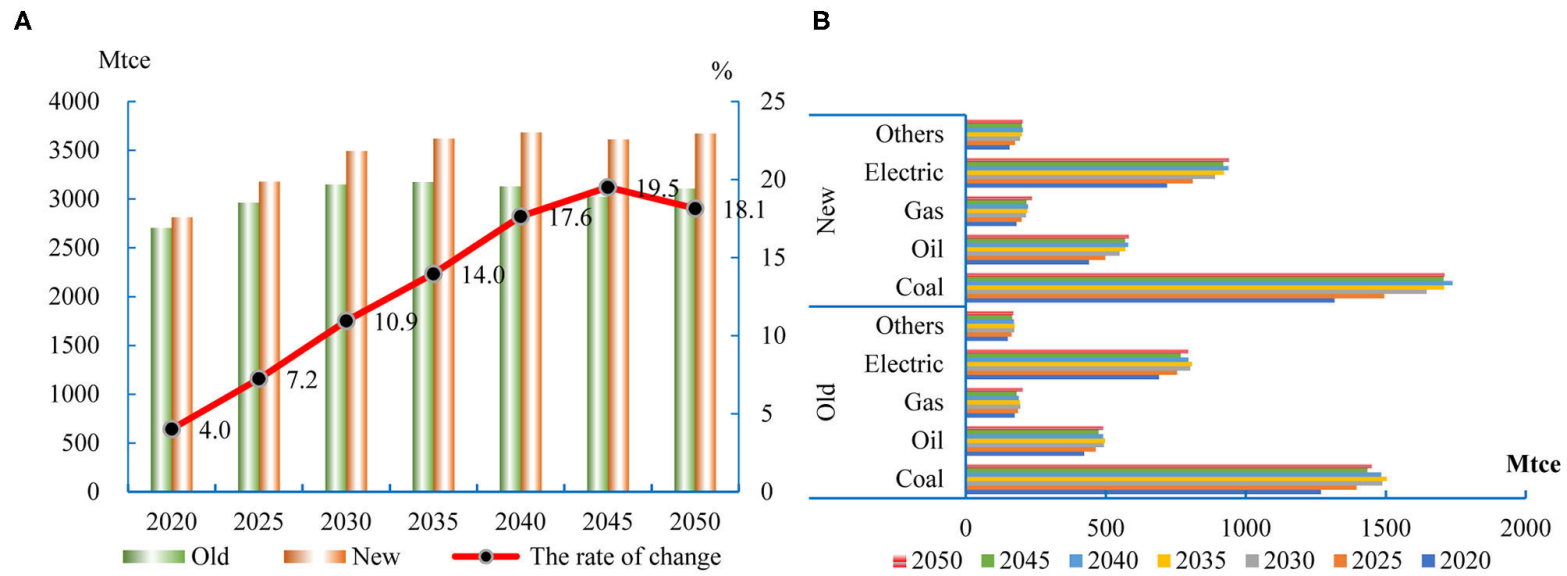
The energy consumption in the industrial and commercial sectors. **(A)** The changes of energy consumption in industrial and commercial sectors. **(B)** The changes of different energy types.

People in different life cycle stages have different consumption patterns (20). The demand for goods and services is affected by the population size and structure. The consumption of goods and services decides the energy input of industrial and commercial sectors. The results of the Input-Output Table Analysis indicate that the impact of the two-child policy on the energy consumption of industrial and commercial sectors changes dynamically over time (Figure 5A). In 2050, the two-child policy will result in an 18.1% increase in energy consumption in the industrial and commercial sectors. The electricity consumption will be the most affected, and the gas will be the least affected (Figure 5B). The growth of energy consumption exceeds the population growth. There are three reasons for the result. Firstly, the two-child policy will promote urbanization, and urban residents have greater consumption expenditure per capita. Second, the two-child policy will mainly increase the number of minors in the short term. Since minors have no income, this will increase the marginal propensity to consume. Third, the increase of the working-age population will promote economic development and increase residential income, which will lead to the increase of total consumption expenditure.

### Energy Consumption in the Passenger and Freight Transport Sectors

**Figure 6:**
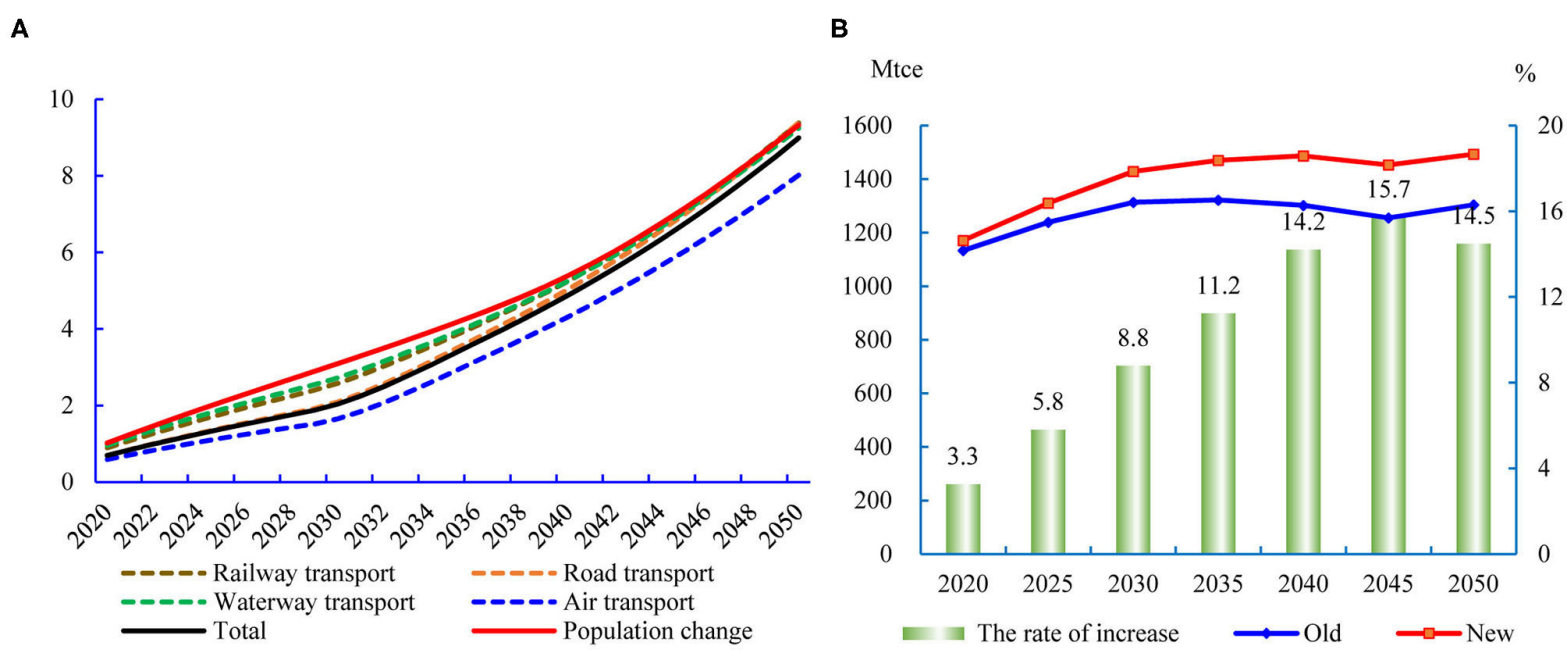
Effect of the two-child policy on energy consumption in the transport sector. **(A)** The changes of energy consumption in the passenger transport sector (%). **(B)** Energy consumption in the freight transport sector.

According to the results in Figure 6A, the two-child policy leads to an increase in energy consumption in the passenger transport, but it is not keeping pace with the population growth. Before 2030, the growth of energy consumption is smaller than population growth, because most of the newborn population are minors. In 2030, the new policy will lead to a 2.04% increase in energy consumption in the passenger transport, which is obviously lower than population growth (3.10%). Then, as the newborn population grows up, the growth rate of energy consumption in the passenger transport is gradually converging with population growth. Given that the minors have a lower travel demand than adults and elders (43), the growth of energy consumption will be lower than population growth before 2050. In 2050, the total energy consumption of the passenger transport sector will be 270 Mtce in the old policy. The changes caused by the two-child policy will increase the energy consumption to 22.3 Mtce. The rate of increase is 9.0%, which is close to the population increase caused by the two-child policy.

The energy consumption of the freight transport sector is closely related to the development of the industrial and commercial sectors. Figure 6B shows the dynamic process of energy consumption in the freight transport sector. In the old birth control policy, the total energy consumption of the freight transport sector in 2050 is expected to be 1,493 Mtce. The new birth policy will increase energy consumption by 189 Mtce. The rate of increase is 14.5% exceeding the population growth.

### Decomposition of Total Energy Consumption

Combined with the energy consumption in the four sectors, the terminal energy consumption will reach 6,107 Mtce in 2050 in Scenario II (the two-child policy is implemented), an increase of 16.2% when compared with Scenario I (Supplementary Figure 5). Since the industrial and commercial sectors accounted for 75.3% of China's final energy consumption, the contribution of the “indirect effect” greatly exceeds the “direct effect” caused by the residential and passenger transport sectors. Finally, the growth of total energy consumption is significantly higher than the population growth. There are two transmission routes of the two-child policy affecting energy consumption. One is that an increase in population size leads to an increase in energy consumption. Another is that the changes in lifestyles change the energy consumption scale and structure. Therefore, the growth of total energy consumption can be decomposed into the impacts of population sizes and lifestyle change. The impacts of lifestyle change are calculated by


(16) $$\begin{array}{r} \left(1+TEC\right)=\left(1+IPS\right)\times \left(1+ILC\right), \end{array}$$

Where,

*TEC* is the growth of total energy consumption.

*IPS* is the growth of population size. It reflects the impact of population size on energy consumption.

*ILC* is the impact of lifestyle changes on energy consumption.

**Figure 7:**
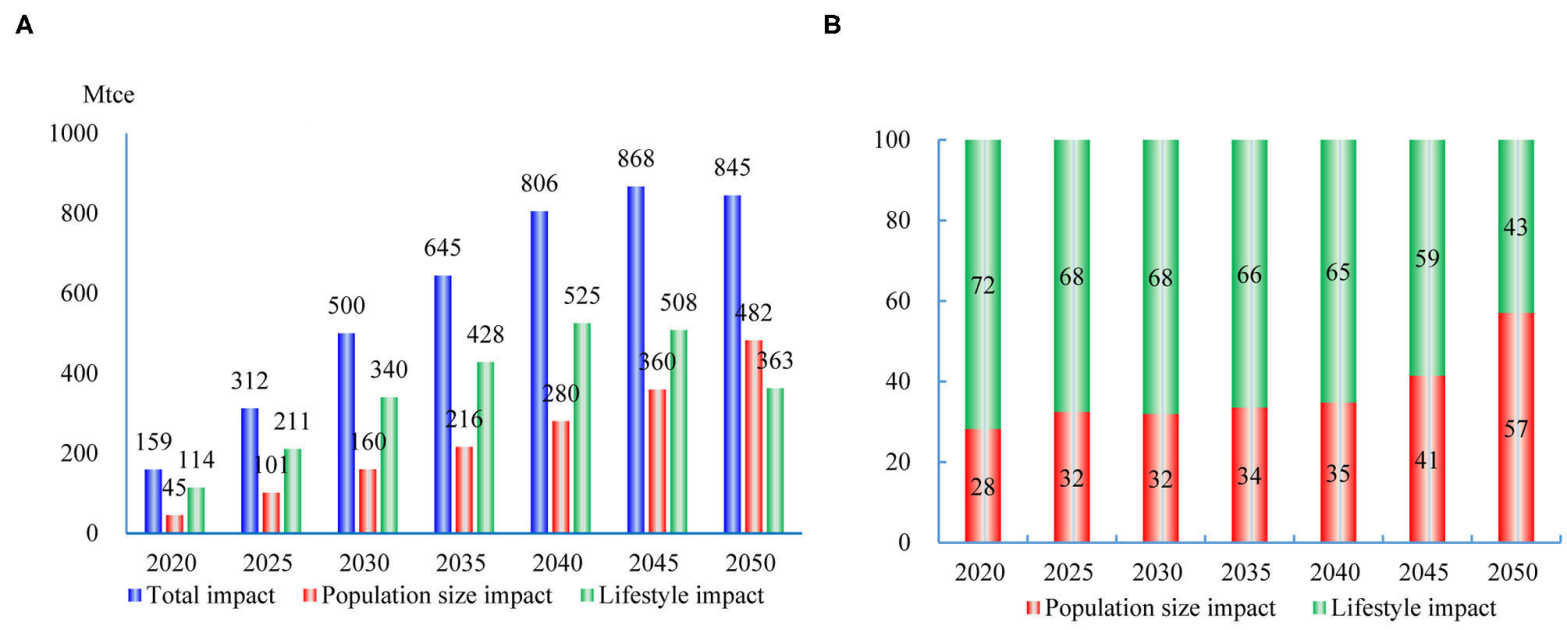
The factor decomposition of the total energy consumption changes. **(A)** The decomposition of total energy consumption changes. **(B)** The contributions of different impact factors to energy consumption (%).

The factor decomposition results show that the contribution of lifestyle changes continues to decline (Figure 7A). In 2020, the two-child policy increases energy consumption by 159 Mtce, in which the change in population size contributes 28%, but the change in people's lifestyle contributes 72%. In 2050, the two-child policy increases energy consumption by 845 Mtce, in which the change in population size contributes 57%, but the contribution of lifestyle change reduces to 43% (Figure 7B). The two-child policy has also changed the energy structure (Supplementary Figure 6). The main reason for these phenomena is that the two-child policy will mainly increase the number of minors in the short term. This makes the impact of the lifestyle changes on energy consumption is greater than the population growth. However, the mediating effect of lifestyle changes will gradually decrease with the newborns growing up.

## Discussion

This article studies the impact of birth control policy on energy consumption from the new perspective of lifestyle changes. The PLE model analyzes the dynamic changes in China's energy consumption from 2020 to 2050. The impacts of the two-child policy on energy consumption in different sectors are as follows. Firstly, different from the existing works of literature (9), this article finds that the REC growing more slowly than population size because of the expansion of household size. In 2050, the two-child policy will lead to a 33.31 Mtce increase in REC, in which the increase in population size contributes 53.67 Mtce and the expansion of household size contributes −20.35 Mtce. Secondly, the two-child policy will increase the marginal propensity to consume and promote economic development (20), resulting in the “indirect effect” on energy consumption in the industrial and commercial sectors and freight transport sector. In 2050, the new policy will increase energy consumption by 18.1% in the industrial and commercial sectors and 14.5% in the freight transport sector, far outpacing a population increase of 9.3%. Thirdly, considering that the travel demand of minors is lower than that of adults and elders (44), the growth of energy consumption in the passenger transport sector is anticipated to be lower than that of the population until 2030. However, in the long term, the growth of passenger transport energy consumption will synchronize with population growth. Synthesizing the “direct effects” and “indirect effects,” the impact of the two-child policy on total energy consumption significantly exceeds population increase when considering the changes in lifestyle. The two-child policy will lead the total energy consumption to increase by 16.2% in 2050, far outpacing the population size increase of 9.3%.

Many studies have held an optimistic attitude toward the future trend of energy consumption and carbon emissions in China (4, 24). The main reason is that the decline in China's population and population aging are becoming increasingly prominent (8, 45). Some works of literature even suggested that the aging population will reduce energy consumption, which is equivalent to the effect of technological change (5, 42). The results of this article, however, show that the previous view is overly optimistic. The two-child policy not only increases the total population but also changes people's lifestyle, which leads to the increase of energy consumption to exceed the expectation. As China is the most populous and the most energy-consuming country (4), the new policy will have a negative impact on global climate change mitigation.

There are three main contributions in this article when compared with the existing literature (4, 16, 23, 46), Firstly, new transmission mechanisms for the impact of birth control policy on energy consumption are proposed. The conventional wisdom holds that the fertility rate affects energy consumption only through the change in population size (8, 9, 12). The people's lifestyle is identified as another significant transmission route in this article. We also find that the impacts of lifestyle changes on energy consumption vary greatly across the four sectors. Secondly, we discover the non-linear relationship between the fertility rate and energy consumption by the dynamic analysis. In traditional static scenario analysis, the dynamic impact of the fertility rate on energy consumption cannot be observed (16, 24). The PLE model considers the changes in people's lifestyles in different life cycle stages and estimates the non-linear influence of the lifestyle changes on energy consumption. Thirdly, this study provides a new framework to predict energy consumption across the world. Energy consumption forecasting is an important prerequisite for energy policy-making. However, the popular models adopted by governments and international organizations ignore the lifestyle change caused by the birth control policy (10, 11). As a result, biased energy consumption forecasting might mislead policy-making. This study will be helpful to improve the traditional energy forecasting models.

Meanwhile, it is important to note that all the predictions are based on the current energy use efficiency. This study has not considered the changes in energy structure and energy intensity resulting from technological progress (47).

## Conclusion and Policy Implications

This article presents the PLE model to study how birth control policy affects people's lifestyle and energy consumption. We find that the trend of energy consumption is significantly different from that predicted by traditional literature when considering the changes in lifestyle. The expansion of household size caused by the two-child policy will reduce REC by 3.42%. The energy consumption will grow more slowly than the population in the passenger transport sector, because the minors have less travel demand than adults and elders. The “indirect effect” (in the industrial, commercial, and freight transport sectors) will result in energy consumption that exceeds population growth. On the whole, the two-child policy will increase total energy consumption by 16.2% in 2050, far outpacing a population increase of 9.3%. In other words, ignoring lifestyle changes will lead to an underestimation of energy consumption. Contrary to conventional optimistic wisdom, we believe that the two-child policy will make it difficult for China to meet promised energy conservation goals, which deserves close attention from Chinese policy makers. This article is the first to fully reveal the transmission mechanism between birth control policy and energy consumption by considering the changes of lifestyle and population size simultaneously. The methods and findings have important implications for global energy consumption forecasting and policy making. Governments and international organizations should pay attention to the changes in lifestyles caused by the new birth control policy.

In conclusion, the two-child policy will change the trend of China's energy consumption and may cause the Chinese government to miss its promise of peaking the energy consumption by 2030 (48). Therefore, the Chinese government needs to implement more effective policies to meet the challenge of population growth. Implementing more stringent energy conservation measures and improving energy efficiency further are the solutions. Based on the above conclusions, the following policy implications are proposed. Firstly, the government needs to reassess the plans for energy production and supply. China's energy supply is controlled by the government and the state-owned enterprises. Because the two-child policy will lead to a larger-than-expected increase in energy consumption in the future, governments need to increase energy production and supply over time. Secondly, the energy sources of REC must be optimized. At present, the proportion of coal consumption in REC is excessively high (35.6 and 3.7% in the rural and urban areas, respectively) (26). Because coal has a lower energy transfer efficiency (37), the “coal to gas” project should be widely implemented in rural areas. Meanwhile, the grown-up minors can be encouraged to live with their parents to make a scale effect and save REC. Thirdly, the industrial and commercial sectors are the main users of energy. It is critical to reduce energy intensity. The feasible measures include improving energy efficiency and reducing the consumption of energy-intensive goods. The Chinese government needs to phase out some energy-intensive industries and equipment that fail to meet energy efficiency standards (49, 50) and encourage people to use energy-efficient products. Finally, improving the share of public transport is an effective approach to reduce transportation energy consumption. The government can develop the shared mobility model in densely populated cities. In rural areas, meeting the travel needs of the elders and minors and reducing the proportion of private trips are reasonable.

## Data Availability Statement

The original contributions presented in the study are included in the article/Supplementary Materials, further inquiries can be directed to the corresponding author.

## Author Contributions

FC and YWu: designed the study. FC and YWa: contributed to data acquisition. WW and YWa: contributed to data analysis. YWa and YWu: wrote the original manuscript. WW and YWu: revised the manuscript. All authors contributed to manuscript revision, read, and approved the submitted version.

## Funding

This work was supported by National Social Science Fund of China (Grant No.18BJY050), China Association of Higher Education (Grant No. 21YDD05), and General Program of National Natural Science Foundation of China (Grant No. 72172020).

## Conflict of Interest

The authors declare that the research was conducted in the absence of any commercial or financial relationships that could be construed as a potential conflict of interest.

## Publisher's Note

All claims expressed in this article are solely those of the authors and do not necessarily represent those of their affiliated organizations, or those of the publisher, the editors and the reviewers. Any product that may be evaluated in this article, or claim that may be made by its manufacturer, is not guaranteed or endorsed by the publisher.

## Footnotes

^1^The household types can be classified by the household size, intergenerational structure, age distribution, and so on.

^2^The existing works of literature classified the household types into the one-generation household, two-generation household, and three-generation household. The two-child policy will increase the number of the youngest generation, but will not change the type of household. Therefore, we cannot classify household types by the intergenerational relationships when studying the impact of the two-child policy on REC.

^3^The website of ProFamy software: http://www.profamy.com.cn/.

^4^The Chinese Sixth National Population Census data are available at: http://www.stats.gov.cn/tjsj/pcsj/rkpc/6rp/indexch.htm.

^5^Data were derived from the Chinese General Social Survey (CGSS) (2017), collected by the Department of Sociology at Renmin University of China. The original data are available at: http://cgss.ruc.edu.cn/index.php?r=index/index&hl=en.

^6^The life cycle is divided into three phases in this article: minors (under 14 years old), adults (15–59 years old), and elders (over 60 years old).

^7^The most recent data are collected in 2018 and are available at: https://opendata.pku.edu.cn/dataverse/CFPS.

^8^The data in 2015 are available at: https://microdata.stats.gov.cn/.

^9^*Human Development Report 2019* forecasted the global urbanization rate was estimated to grow to nearly 70% by 2050.

^10^Here is a comparison with the old fertility policy, hereinafter.
