# Supplementary material for: How Two-Child Policy Affects China's Energy Consumption: The Mediating Role of Lifestyle
Source: Front Public Health. 2022 Apr 6;10:866324. doi: 10.3389/fpubh.2022.866324 (PMC9019122; doi:10.3389/fpubh.2022.866324)
Supplement: Supplementary file 1 [file Data_Sheet_1.docx]

**How Two-Child Policy Affect China’s Energy Consumption: the Mediating Role of Lifestyle**

**Supplementary Table 1| Energy consumption index for different household types**

| Area | Household size | Coal | Oil | Gas | Electric | Other |
| --- | --- | --- | --- | --- | --- | --- |
| Urban | 1 | 100.00 | 100.00 | 100.00 | 100.00 | 100.00 |
|  | 2 | 134.48 | 167.84 | 193.72 | 146.63 | 140.89 |
|  | 3 | 153.20 | 198.65 | 203.31 | 173.45 | 147.64 |
|  | 4 | 171.83 | 221.54 | 223.93 | 192.95 | 155.92 |
|  | 5 | 182.95 | 224.90 | 265.84 | 264.51 | 168.74 |
|  | 6 | 188.73 | 249.41 | 301.52 | 290.93 | 179.95 |
|  | 7+ | 190.64 | 276.42 | 314.49 | 340.37 | 181.67 |
| Rural | 1 | 100.00 | 100.00 | 100.00 | 100 | 100.00 |
|  | 2 | 127.87 | 122.20 | 147.17 | 147.99 | 138.96 |
|  | 3 | 130.78 | 214.78 | 159.11 | 170.52 | 141.51 |
|  | 4 | 136.58 | 231.12 | 173.53 | 216.06 | 150.57 |
|  | 5 | 144.62 | 273.38 | 194.74 | 242.15 | 176.02 |
|  | 6 | 171.30 | 292.92 | 206.84 | 274.31 | 184.75 |
|  | 7+ | 196.16 | 313.40 | 231.34 | 307.87 | 208.08 |

**Supplementary Table 2. Parameter assumptions for industrial system**

| Year | Propensity to consume | | [GDP per-worker](https://www.baidu.com/link?url=fwRjrHfE62inP9nS08lkexvnkLCtc8oxqQWgrK2bTpfq0Kpl2iUvMcoMMmMgCUmAg3maB2NoyW1NSkZ2W8oX_RZGomX3L_SfeYO-e42YAEa&wd=&eqid=ec6de8cd00005552000000035eb94752)  (10,000CNY) |
| --- | --- | --- | --- |
|  | Old policy | New policy |  |
| 2020 | 38.1% | 39.2% | 9.42 |
| 2025 | 38.3% | 39.8% | 12.88 |
| 2030 | 38.5% | 40.4% | 17.21 |
| 2035 | 38.8% | 41.0% | 22.53 |
| 2040 | 39.0% | 41.7% | 28.93 |
| 2045 | 39.3% | 42.3% | 36.48 |
| 2050 | 40.5% | 43.0% | 45.23 |

Note: [GDP per-worker](https://www.baidu.com/link?url=fwRjrHfE62inP9nS08lkexvnkLCtc8oxqQWgrK2bTpfq0Kpl2iUvMcoMMmMgCUmAg3maB2NoyW1NSkZ2W8oX_RZGomX3L_SfeYO-e42YAEa&wd=&eqid=ec6de8cd00005552000000035eb94752) which is estimated according to the EIA prediction(Energy Information Administration, 2019). The propensity to consume is estimated according to the experience of European Union Nations(York, 2007).

**Supplementary Figure 1 |** The trend of energy consumption for standardized household.

**Supplementary Figure 2 |** the trend of *PCI* for different goods and services

**Supplementary Figure 3 |** Household consumption expenditure in 2050 (Unit: 1 million CNY).

Note: we estimated the impact of two-child policy on the final consumption expenditure and intermediate input for 149 industries every 5 years in 2020-2050. This figure shows only the results in 2050.

**Supplementary Figure 4 |** The travel index for different lifecycle phase.

Note: the original data is derived from Chinese Urban Household Survey (CUHS) database in 2015.

**Supplementary Figure 5 |** The trend of total energy consumption

**Supplementary Figure 6 |** The change of energy structure in different policies (Unit: Mtce).
